# pH dependence of the chirality of nematic cellulose nanocrystals

**DOI:** 10.1038/s41598-019-47834-w

**Published:** 2019-08-05

**Authors:** Chenxi Li, Julian Evans, Nan Wang, Tingbiao Guo, Sailing He

**Affiliations:** 1 Centre for Optical and Electromagnetic Research, Zhejiang University, Hangzhou, 310058 China; 2 Department of Electromagnetic Engineering, Royal Institute of Technology, S-100 44 Stockholm, Sweden

**Keywords:** Bioinspired materials, Liquid crystals, Colloids

## Abstract

Cellulose nanocrystals produced by acid hydrolysis of native cellulose form a well-known chiral nematic liquid crystal phase. The mechanism involved in the formation of chirality has been the subject of a vigorous discussion. The pH and concentration dependence of the phase is studied using cellulose nanocrystal droplets within a silicon oil suspension, which allows for convenient real-time microscale manipulation of phase behaviors and properties. We demonstrate the existence of nematic phases at both low and high pH regions consistent with the Stroobants - Lekkerkerker - Odijk theory. Our results confirm electrostatic interactions play a critical role in controlling the strength of the chirality.

## Introduction

Cellulose, the world’s most abundant biopolymer, has attracted widespread interest throughout history for wood, paper, cloth technologies and much more modern design materials^1,2^. Cellulose nanocrystals (CNCs) from hydrolyzing native cellulose generally present a rod-like shape with the negative charge surface^3^ and form liquid crystals (LCs) above a critical concentration^4^. CNCs are well known for forming the chiral nematic phase LCs that could preserve the helical orientation in solid films^5,6^ and serve as a template for chiral structures composed of other materials^7–10^. Generally speaking, the three factors, which determine the phase behavior of CNC LCs, are starting material, concentration and pH^11^. Starting material allows for a good amount of control over the dimensions of CNCs. Cotton^12^, wood^13^, and other higher plants^14,15^ allow for the production of CNCs with the average length that can be tuned between 100 nm and 500 nm with high polydispersity depending on the material^16^, while bacterial CNCs are typically a few microns long^17^. It is more difficult for higher plant CNCs to form nematic phase LCs compared with bacterial CNCs as their aspect ratios are smaller. Changing the concentration of CNCs has been shown to tune the chiral nematic pitch over approximately an order of magnitude^18,19^. pH allows for control of the ionic strength of CNC suspensions, which determines the Debye length and surface charges on each CNC^20^. Adding salt to a solution allows for controlling of the ionic strength without altering the charge on each rod^21^. Previous reports demonstrated that reducing the Debye length allowed for a slight reduction in the pitch at low salt concentration and produced a “tactoidal system with divergent pitch” above a critical concentration^22^. In Stroobants - Lekkerkerker - Odijk (SLO) theory, the twist parameter becomes significantly smaller as the Debye length is reduced, below the size of the typical ion promoting the nematic phase over the chiral nematic phase^23^. In Derjaguin-Landau-Verwey-Overbeek (DLVO) theory, the van der Waals force between two rods is generally attractive and promotes a parallel orientation while the electrostatic force between rod-like polyelectrolytes promotes a perpendicular orientation^24^. Some researchers have hypothesized the chirality derived from the helical intrinsic twist in nanocrystals^25^ while some assumed there was a chiral charge distribution on the CNC surface after acid hydrolysis^23^, so the chiral origin of CNC LC system is still a debated question^26^.

In the present work, we prepared and characterized CNCs using established protocols, encapsulated them in silicon oil suspension and tuned the pH between 1 and 12 using 9.7 wt% sulfuric acid and 1 M sodium hydroxide. CNC LCs with tunable pH and fixed concentration showed two nematic phases in low and high pH regions. This loss of chirality in CNC LCs corresponds to a reduced Debye length and a mitigated Coulombic repulsion. The effect of electrostatic forces in CNC suspensions was analyzed and experimental results were in accordance with the theoretical analysis.

## Results

### Phase transition

**Figure 1:**
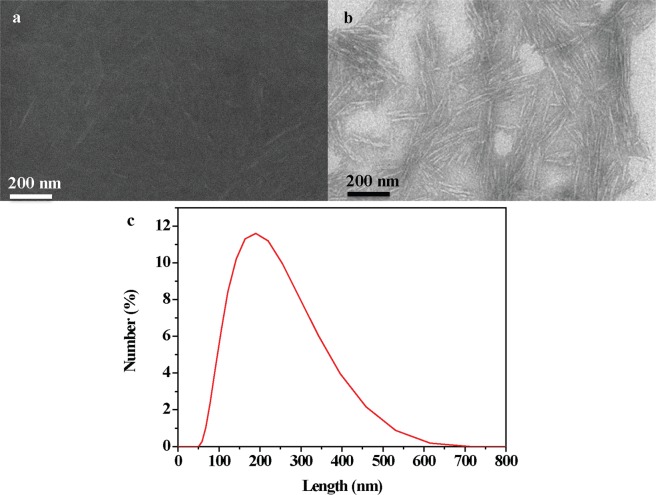
SEM (**a**) and TEM (**b**) images of the CNCs. Length distribution of the CNCs (**c**) measured by dynamic laser scattering.

**Figure 2:**
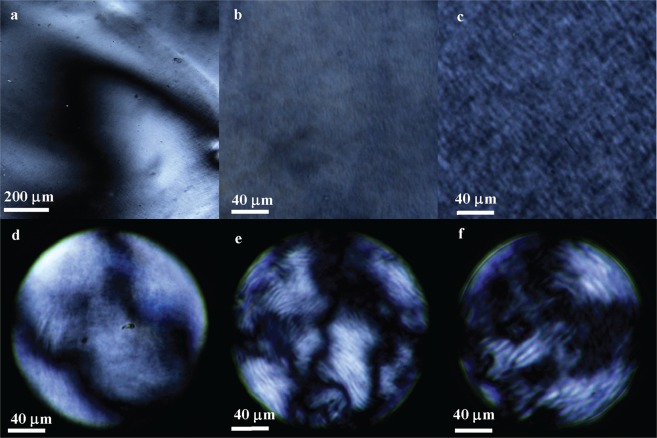
POM images of CNC LCs. Nematic phase CNC LCs (pH = 1.5) in bulk (**a**) and droplet surrounded by silicone oil (**d**). Small pitch chiral nematic LCs (pH = 2) in bulk (**b**) and droplet (**e**). Long pitch chiral nematic phase LCs (pH = 3) in bulk (**c**) and droplet (**f**). Estimating CNC concentration for these LCs is 13 wt%.

The morphological characterization of CNCs after sulfuric acid hydrolysis (Fig. 1) is typical of these well-studied materials. Scanning electron microscope (SEM) and transmission electron microscope (TEM) images of CNCs indicate CNCs have a dimension around 5–20 nm thick and 100–200 nm long consistent with previous reports^12,27^. The length distribution of the CNCs is also measured by dynamic laser scattering, which demonstrates that the well separated individual rod-like particles are around 200 nm long (Fig. 1c). POM images of CNC LCs in bulk exhibit the nematic (Fig. 2a) and chiral nematic phase (Fig. 2b,c) with the typical Schlieren and fingerprint texture respectively. CNC LCs encapsulated in silicone oil droplets (Fig. 2d–f) allows for convenient real-time study of phase behaviors of cellulose dispersions. Silicone oil plays no noticeable role in the phase behavior itself and CNC LC phase behaviors discussed here can be observed in the bulk with no silicone oil present (Fig. 2a–c) or the droplet form (Fig. 2d–f) with same conditions, when the diameter of CNC-silicone oil droplet is above 100 μm^28^. The initial preparation of chiral nematic CNC LC has a pH around 2 after dialysis, and the Zeta potential of CNC LCs is around −26 mV, which is below the threshold for agglomeration in CNC systems^29,30^.

**Figure 3:**
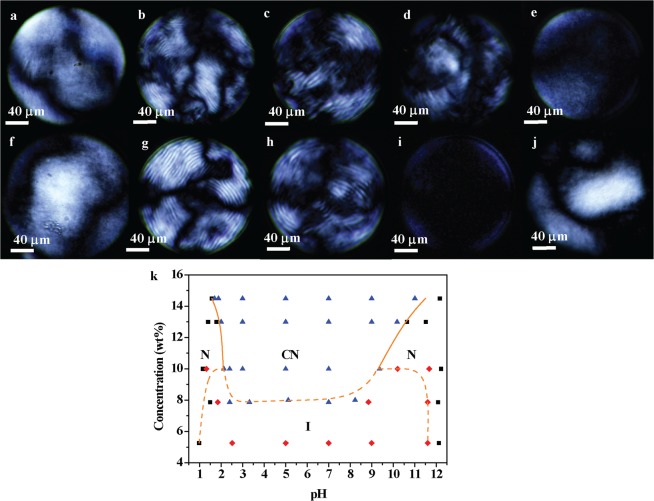
POM images of 13 wt% CNC LC in droplets (**a**–**e**): (**a**) pH = 1.5, (**b**) pH = 2, (**c**) pH = 3, (**d**) pH = 5, (**e**) pH = 10. POM images of 8 wt% CNC LC in droplets (**f**–**j**): (**f**) pH = 1.5, (**g**) pH = 2, (**h**) pH = 9, (**i**) pH = 10, (**j**) pH=12. Phase diagram of CNC LCs are shown in (**k**), where letter “N”, “CN”, and “I” represent nematic (black squares), chiral nematic (blue triangles) and isotropic phases (red diamonds) respectively.

POM images of CNC LC droplets are shown in Fig. 3a–j and the phase diagram is presented in Fig. 3k. At a high concentration of 13 wt% (Fig. 3a–e), there exists two nematic phases at pH = 1.5 (Fig. 3a) and pH = 10 (Fig. 3e), showing the typical Schlieren texture. When pH is around 2, 3 and 5, CNCs form chiral nematic phase LCs with fingerprint textures (Fig. 3b–d). There shows a sharp transition from nematic to chiral nematic phases at both low and high pH boundaries (Fig. 3k). When the concentration of CNC LCs is lowered to 8 wt% (Fig. 3f–j), there is a slightly different sequence phase behaviors compared with 13 wt% CNC LCs. There is an isotropic phase (Fig. 3i) between the chiral nematic (Fig. 3h) and nematic phase (Fig. 3j), showing full dark with no birefringence under the POM, which means CNCs are randomly oriented without any LC order. 5 wt% CNC suspensions exhibit the isotropic phase at pH ranging from 1.5 to 11, and form the nematic again when pH below 1.5 or above 11 (Fig. 3k). Observing five batches of CNC suspensions with different concentrations (14.5 wt%, 13 wt%, 10 wt%, 8 wt%, and 5 wt%) and tunable pH (1~12), we obtain the CNC LC phase diagram describing phase transitions among isotropic, nematic and chiral nematic phase (Fig. 3k). Black squares, blue triangles and red diamonds in Fig. 3 represent nematic, chiral nematic and isotropic phases respectively, where each point is deduced from the LC texture of 20 droplets observed with POM. In Fig. 3k, there are two nematic phase regions for pH below 2 or above 10, and chiral nematic above 8 wt% when the pH ranges from 2 to 10. The isotropic phase appears for 5 wt% CNC suspensions in pH between 2 and 9 and appears for concentrations up to 10 wt% in two windows for pH = 1.5~2 and pH = 9~11.

### Pitch

POM images of chiral nematic CNC LCs (Fig. 3b–d,g,h) indicate that pH and concentration play a crucial role in pitch. 13 wt% CNC suspensions form a short pitch chiral nematic phase at pH = 2 (Fig. 3b), while at pH = 3 it is uniform chiral nematic phase with the pitch visually longer than at pH = 2 (Fig. 3c). At pH = 5 there is a mixture of long-pitch domains and domains with no noticeable chiral nematic character (Fig. 3d). 8 wt% CNC LCs form chiral nematic phases with the similar long pitch at the pH = 2 (Fig. 3g) and pH = 9 (Fig. 3h), which is reasonable since the interparticle spacing is significantly larger than the Debye length^18^. 8 wt% chiral nematic CNC LCs (Fig. 3g) present a longer pitch compared with 13 wt% CNC LCs (Fig. 3b) at the same pH around 2, since lower particle concentrations allow for larger interparticle spacing and promotes the longer pitch^11,19^.

## Discussion

**Figure 4:**
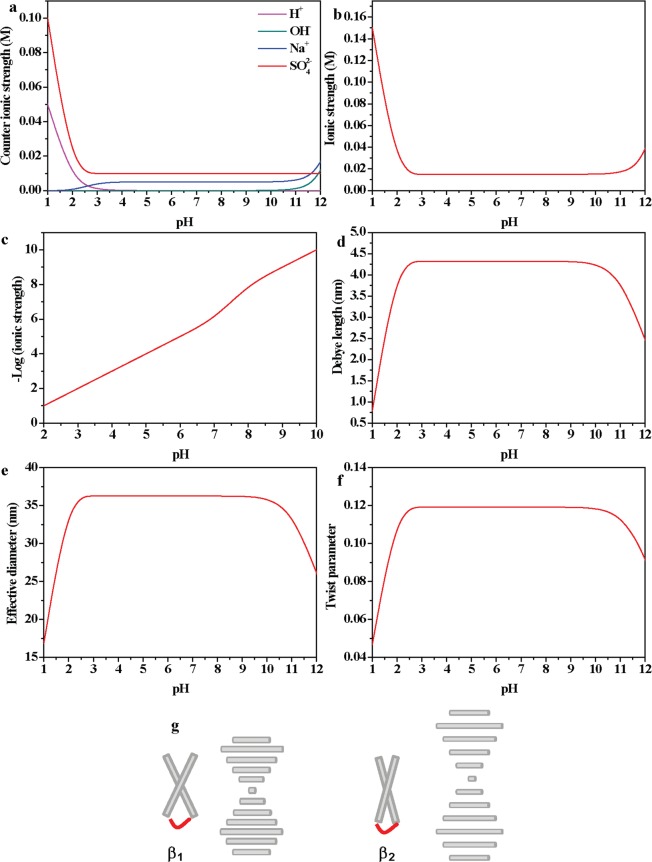
Calculated ionic strength of H^+^, OH^−^, Na^+^ and (**a**) and the ionic strength *I* of CNC suspensions as a function of the pH (**b**), where ionic strength *I* was increased slightly at pH = 2~10 shown in (**c**). Calculated Debye length *λ*_D_ (**d**), effective diameter *D*_eff_ (**e**) and twist parameter *h* (**f**) as a function of the pH. (**g**) Schematic of the chiral nematic phase with large displacement angle *β*_1_ with small pitch ${{\rm{p}}}_{1}=2{\rm{\pi }}d/{{\rm{\beta }}}_{1}$ (left), and chiral nematic phase with small displacement angle *β*_2_ with long pitch ${{\rm{p}}}_{2}=2{\rm{\pi }}d/{{\rm{\beta }}}_{2}$ (right). As the displacement angle *β* decreased (${{\rm{\beta }}}_{1} > {{\rm{\beta }}}_{2}$), the pitch increased (${{\rm{p}}}_{1} > {{\rm{p}}}_{2}$).

The phase behavior of the rod-like suspension is determined by the particle concentration and the electrostatic properties of the solution. The four ions present in solution are H^+^, OH^−^, Na^+^ and ${{\rm{SO}}}_{4}^{2-}$ and the corresponding molar concentration *c* can be calculated from the experimental procedure. The ionic strengths of H^+^, OH^−^, Na^+^ and ${{\rm{SO}}}_{4}^{2-}$ are calculated by $\frac{1}{2}c{z}^{2}$, where *z* donates the charge number and calculated counter ionic strengths are shown in Fig. 4a. Total ionic strength *I* (Fig. 4b) of CNC suspensions is calculated as $I=\frac{1}{2}\sum _{i=1}^{n}\,({c}_{i}{z}_{i}^{2})$ and rises sharply at pH below 2 and above 11 near the nematic phase boundary (Fig. 3k). Theoretical Debye length ${\lambda }_{D}={(\frac{2{e}^{2}I}{\varepsilon {\varepsilon }_{0}{K}_{B}T})}^{-1/2}$ is shown in Fig. 4d, where *λ*_D_ is relatively a constant (*λ*_D_ ~ 4.25 nm) for pH ranging from 2 to 10 while decreases rapidly when the pH goes below 2 or above 10. The electrostatic force between rod-like polyelectrolytes promotes a perpendicular orientation, and the expected phase behavior is characterized by a twist parameter *h*, which is the ratio of the Debye length *λ*_D_ and effective diameter *D*_eff_ of rods^18,31^. The effective diameter *D*_eff_ of a charged rod is ${D}_{eff}=D+5.54{\lambda }_{D}$ in CNC suspensions, where *D* denotes the diameter of CNC rods^32^. Taking *D* = 12.5 nm from the SEM (Fig. 1a) and TEM (Fig. 1b) images, the calculated *D*_eff_ (Fig. 4e) shows a relatively constant *D*_eff_ (around 37 nm) at pH = 2~10 and decreases rapidly when the pH goes below 2 and above 10. The twist parameter *h* is calculated by *h* = *λ*_*D*_/*D*_*eff*_ (Fig. 4f) that is also relatively constant (*h*~0.12) at pH ranging from 2 to 10 while decreasing sharply when pH goes below 2 or above 10. CNC LCs have a low Debye length below 2 nm (Fig. 4d), and the electrostatic twist is weakened (Fig. 4f), thus the nematic phase forms at pH below 2 and above 10 (Fig. 3a,e). At pH between 2 and 10, the large electrostatic twist (Fig. 4f) promotes the chiral nematic phase (Fig. 3b–d) over the nematic phase (Fig. 3a,e). In SLO theory, the electrostatic twist is the largest when *λ*_D_ is on the order of the polyion diameter while the electrostatic twist diminishes rapidly when the Debye length below the order of the polyion diameter^23^. CNCs have sulfate half ester on their surfaces after sulfuric acid hydrolysis and the second pKa of sulfuric acid is 1.99^3,33^, the grafted ester groups should be predominantly protonated below pH = 2 and negatively ionic at higher pH. The phase transition of 13 wt% CNC suspensions between nematic and chiral nematic phase LCs is consistent with SLO theory. Surprisingly, there are isotropic phases between nematic and chiral nematic phase at pH around 1.5 and 10 for 8 wt%~10 wt% CNC suspensions (Fig. 3k). As the Debye length begins to decrease the chirality disappears quickly and the effective diameter *D*_eff_ is almost three times larger than the physical *D* (Fig. 4e), so the Onsager criterion (The volume fraction $\phi  \sim 4{D}_{eff}/L$ and *L* is the length of CNC rods) is harder to reach due to a lower effective aspect ratio. In Onsager theory, the phase separation of isotropic and LC phases is based on the entropic steric interactions and excluded volume^34^. Once the Debye Length is minimized the effective diameter is slightly larger than the physical diameter and the Onsager criterion is more easily met. The transition between nematic and chiral nematic is primarily dictated by *h*, where electrostatic interactions play a critical role in chiral helicoidal ordering in the high concentration region.

The Frank-Elastic Energy for twisting is $1/2{K}_{22}{(\nabla \times \nabla \times n)}^{2}$, where *K*_22_ is the twist elastic parameter and *n* donates the director of LCs, thus there is an entropic force that is quadratic in the displacement angle for all angles. In the chiral nematic phase region with large Debye length, considering the electrostatic interaction between a pair of charged rods we get a small angle approximation that is quadratic in the displacement angle *β* (Fig. 4g). When only considering a pair-wise interaction, the Coulombic energy is minimized for orthogonal rods. Where the interparticle separation *d* satisfies *d* = *Lβ*, the interaction with the next nearest neighbor mitigates the savings associated with rotation thus enforcing an effective minimum pitch. The pitch *p* of the chiral nematic phase is *p* = 2*πd*/*β*, combining the equation (*d* = *Lβ*), we get the minimum pitch should be *p* = 2π*L* on the scale of a few microns, which is consistent with previous reports^18,19^. In chiral nematic regions with pH ranging from 2 to 10, there shows an increased ionic strength (Fig. 4c) and electrostatic forces decay faster and thus the twist interaction is decreased and the displacement angle *β* between CNCs becomes smaller (Fig. 4g), leading to a larger pitch (Fig. 3b–d).

## Methods

### Preparation of CNCs

CNCs were prepared by the established hydrolysis protocols^35^. 6.5 g of degreasing cotton was dispersed in 70 mL of 65 wt% sulfuric acid and stirred at 46 °C in a water bath for one hour. 70 mL of deionized water was added to the resulting dispersion to quench the acid hydrolysis process. The cellulose mixture was then centrifuged in 1.5 mL tubes at 9000 rpm for 10 minutes around 5 times. This raw product was dialyzed (MWCO 12000) under static conditions for several days until the water pH reached seven, followed by 13500 rpm centrifugation for 40 minutes to purify cellulose dispersion and remove surplus deionized water to obtain CNCs that form chiral nematic LCs with pH and concentration around 2 and 14.5 wt% respectively.

### Phase behaviors and properties

Deionized water was added into the 14.5 wt% CNC suspension to prepare four batch CNC LCs with the different concentration (13 wt%, 10 wt%, 8 wt%, and 5 wt%). Sulfuric acid (9.7 wt%) or sodium hydroxide (1 M) solution were added into 200 μL CNC LCs to make the desired pH, where the mass of adding sulfuric acid or sodium hydroxide solutions was less than 1 wt%, leading to tuning the pH without significantly changing the concentration. CNC LCs was dispersed into silicone oil and stirred for several minutes until homogeneous mixing to get relatively uniform CNC LC droplets. The pH of CNC dispersions was tracked using the pH meter (PH5S) with resolution ratio of 0.01. The concentration of CNC suspensions were determined by weighing 200 μL of CNC suspension with an analytical balance (Mettler Toledo) followed by drying at 40 °C for 48 h to remove the solvent completely and weighing again.

### Characterization and imaging

30 μL 1.5 wt% cellulose dispersions were deposited on the ITO (indium tin oxide) glass by spin-coating at 6500 rpm for 59 s and observed under the SEM (Raith 150 TWO) at a 5 kV accelerating voltage. 0.003 wt % cellulose suspensions were dropped on copper grids, negatively stained with uranyl acetate, and observed by TEM (JEM-1200). Length distribution of the CNCs and Zeta potential of CNC LCs were measured using dynamic laser scattering (Zano ZS). POM images of CNC LCs were taken using POM (Olympus BX-53M) with a rotating stage to observe the LC textures and phase behaviors.

## Conclusion

We were able to tune the pH of CNC LC droplets surrounded by silicone oil at a fixed concentration from pH = 1 to pH = 12 and observed two nematic phases at low and high pH. We demonstrated electrostatic interactions played a critical role in chiral helicoidal ordering. Careful consideration of phase transitions of CNC suspensions showed that the phase behavior was consistent with the theory of charged rigid rods. Improved understanding of the physical mechanisms involved in CNC LC phase behaviors will inspire improved methods for producing templated and functional materials using CNC LC ordering.

## References

[1] Habibi Y, Lucia LA, Rojas OJ (2010). Cellulose Nanocrystals: Chemistry, Self-Assembly, and Applications. Chem. Rev..

[2] Guo X, Wu Y, Xie X (2017). Water vapor sorption properties of cellulose nanocrystals and nanofibers using dynamic vapor sorption apparatus. Sci. Rep..

[3] Shafeiei-Sabet S, Hamad WY, Hatzikiriakos SG (2013). Influence of degree of sulfation on the rheology of cellulose nanocrystal suspensions. Rheol. Acta..

[4] Dong XM, Revol J-F, Gray DG (1998). Effect of microcrystallite preparation conditions on the formation of colloid crystals of cellulose. Cellulose.

[5] Zhao TH (2019). Printing of Responsive Photonic Cellulose Nanocrystal Microfilm Arrays. Adv. Funct. Mater..

[6] Park JH (2014). Macroscopic Control of Helix Orientation in Films Dried from Cholesteric Liquid-Crystalline Cellulose Nanocrystal Suspensions. ChemPhysChem.

[7] Shopsowitz KE, Qi H, Hamad WY, MacLachlan MJ (2010). Free-standing mesoporous silica films with tunable chiral nematic structures. Nature.

[8] Shopsowitz KE, Stahl A, Hamad WY, MacLachlan MJ (2012). Hard templating of nanocrystalline titanium dioxide with chiral nematic ordering. Angew. Chem..

[9] Giese M, Blusch LK, Khan MK, MacLachlan MJ (2015). Functional materials from cellulose-derived liquid-crystal templates. Angew. Chem..

[10] Schlesinger M, Giese M, Blusch LK, Hamad WY, MacLachlan MJ (2015). Chiral nematic cellulose-gold nanoparticle composites from mesoporous photonic cellulose. Chem. Commun..

[11] Lagerwall JPF (2014). Cellulose nanocrystal-based materials: from liquid crystal self-assembly and glass formation to multifunctional thin films. NPG. Asia. Materials..

[12] Campbell, G. M., Liu, Q., Sanders, A., Evans, S. J. & Smalyukh, I. I. Preparation of Nanocomposite Plasmonic Films Made from Cellulose Nanocrystals or Mesoporous Silica Decorated with Unidirectionally Aligned Gold Nanorods. *Materials* **7** (2014). 10.3390/ma7043021 PMC5453334 28788604

[13] Salajková M, Berglund LA, Zhou Q (2012). Hydrophobic cellulose nanocrystals modified with quaternary ammonium salts. J. Mater. Chem..

[14] Habibi Y (2008). Bionanocomposites based on poly(ε-caprolactone)-grafted cellulose nanocrystals by ring-opening polymerization. J. Mater. Chem..

[15] Helbert W, Cavaillé JY, Dufresne A (1996). Thermoplastic nanocomposites filled with wheat straw cellulose whiskers. Part I: Processing and mechanical behavior. Polym. Composites..

[16] Zhang R, Liu Y (2018). High energy oxidation and organosolv solubilization for high yield isolation of cellulose nanocrystals (CNC) from Eucalyptus hardwood. Sci. Rep..

[17] Araki J, Kuga S (2001). Effect of Trace Electrolyte on Liquid Crystal Type of Cellulose Microcrystals. Langmuir.

[18] Schütz C (2015). Rod Packing in Chiral Nematic Cellulose Nanocrystal Dispersions Studied by Small-Angle X-ray Scattering and Laser Diffraction. Langmuir.

[19] Pan J, Hamad W, Straus SK (2010). Parameters Affecting the Chiral Nematic Phase of Nanocrystalline Cellulose Films. Macromolecules.

[20] Kuo T-C, Sloan LA, Sweedler JV, Bohn PW (2001). Manipulating Molecular Transport through Nanoporous Membranes by Control of Electrokinetic Flow:  Effect of Surface Charge Density and Debye Length. Langmuir.

[21] Dong XM, Gray DG (1997). Effect of Counterions on Ordered Phase Formation in Suspensions of Charged Rodlike Cellulose Crystallites. Langmuir.

[22] Hirai A, Inui O, Horii F, Tsuji M (2009). Phase Separation Behavior in Aqueous Suspensions of Bacterial Cellulose Nanocrystals Prepared by Sulfuric Acid Treatment. Langmuir.

[23] Stroobants A, Lekkerkerker HNW, Odijk T (1986). Effect of electrostatic interaction on the liquid crystal phase transition in solutions of rodlike polyelectrolytes. Macromolecules.

[24] Autumn K (2006). Van der Waals Forces: A Handbook for Biologists, Chemists, Engineers, and Physicists. By V Adrian Parsegian. Q. Rev. Biol..

[25] Hanley SJ, Revol J-F, Godbout L, Gray DG (1997). Atomic force microscopy and transmission electron microscopy of cellulose from Micrasterias denticulata; evidence for a chiral helical microfibril twist. Cellulose.

[26] Wang, P. X., Hamad, W. Y. & MacLachlan, M. J. Structure and transformation of tactoids in cellulose nanocrystal suspensions. *Nat Commun* **7** (2016). 10.1038/ncomms11515 PMC4857480 27143197

[27] Elazzouzi-Hafraoui S (2008). The Shape and Size Distribution of Crystalline Nanoparticles Prepared by Acid Hydrolysis of Native Cellulose. Biomacromolecules.

[28] Li Y (2016). Colloidal cholesteric liquid crystal in spherical confinement. Nat. Commun..

[29] Bardet R, Belgacem N, Bras J (2015). Flexibility and Color Monitoring of Cellulose Nanocrystal Iridescent Solid Films Using Anionic or Neutral Polymers. ACS. Appl. Mater. Inter..

[30] He Y-D (2018). Biomimetic Optical Cellulose Nanocrystal Films with Controllable Iridescent Color and Environmental Stimuli-Responsive Chromism. ACS. Appl. Mater. Inter..

[31] Buining PA, Philipse AP, Lekkerkerker HNW (1994). Phase Behavior of Aqueous Dispersions of Colloidal Boehmite Rods. Langmuir.

[32] Dong XM, Kimura T, Revol J-F, Gray DG (1996). Effects of Ionic Strength on the Isotropic−Chiral Nematic Phase Transition of Suspensions of Cellulose Crystallites. Langmuir.

[33] Reid MS, Villalobos M, Cranston ED (2017). Benchmarking Cellulose Nanocrystals: From the Laboratory to Industrial Production. Langmuir.

[34] Onsager L (1949). The Effects of Shape on The Interaction of Colloidal Particles. Ann. NY. Acad. Sci..

[35] Liu Q, Campbell MG, Evans JS, Smalyukh II (2014). Orientationally ordered colloidal co-dispersions of gold nanorods and cellulose nanocrystals. Adv Mater.

